# Targeting exercise-related genes and placental growth factor for therapeutic development in head and neck squamous cell carcinoma

**DOI:** 10.3389/fphar.2024.1476076

**Published:** 2024-10-04

**Authors:** Qingyuan Shi, Haiyue Ying, Weibin Weng

**Affiliations:** Ningbo NO.2 hospital, Ningbo, China

**Keywords:** head and neck squamous cell carcinoma, exercise-related genes, prognosis, bioinformatics, immune microenvironment, placental growth factor (PIGF), drug sensitivity, post-translational modifications (PMTs)

## Abstract

**Background:**

Human cancers, including head and neck squamous cell carcinoma (HNSCC), are complex and heterogeneous diseases driven by uncontrolled cell growth and proliferation. Post-translational modifications (PTMs) of proteins play a crucial role in cancer progression, making them a promising target for pharmacological intervention. This study aims to identify key exercise-related genes with prognostic value in HNSCC through comprehensive bioinformatics analysis, with a particular focus on the therapeutic potential of placental growth factor (PIGF).

**Methods:**

Transcriptome data for HNSCC were obtained from The Cancer Genome Atlas (TCGA) database. Differently expressed genes (DEGs) were identified and analyzed for their prognostic significance. Exercise-related gene sets were retrieved from the Gene Set Enrichment Analysis (GSEA) database. Functional enrichment analyses, including Gene Ontology (GO), Kyoto Encyclopedia of Genes and Genomes (KEGG), and GSEA, were conducted. The biological functions and clinical implications of key genes were further explored through single-gene expression analysis, immune infiltration analysis, and *in vitro* cellular experiments.

**Results:**

The study identified exercise-related genes associated with survival prognosis in HNSCC. GO and KEGG pathway analyses highlighted the biological functions of these genes, and Kaplan-Meier survival curves confirmed their prognostic value. PIGF expression analysis using TCGA data showed its diagnostic potential, with higher expression linked to advanced tumor stages. Single-cell sequencing revealed PIGF’s role in the tumor microenvironment. *In vitro* experiments demonstrated that PIGF plays a pivotal role in enhancing cell proliferation and colony formation in HNSCC, with PIGF knockdown significantly impairing these functions, highlighting its importance in tumor growth regulation. Additionally, PIGF’s predictive performance in drug sensitivity across cancer datasets suggests its potential as a pharmacological target, offering opportunities to modulate the immune microenvironment and improve therapeutic outcomes in cancer treatment.

**Conclusion:**

This study provides new insights into the molecular mechanisms underlying HNSCC and identifies exercise-related genes, particularly PIGF, as promising biomarkers for clinical treatment and personalized medicine. By focusing on PTMs and their role in cancer progression, our findings suggest that targeting PIGF may offer innovative therapeutic strategies.

## 1 Background

Head and neck squamous cell carcinoma (HNSCC) encompasses malignant tumors in regions such as the oral cavity, larynx, and nasopharynx (Porcheri and Mitsiadis, 2021; Howard et al., 2012). According to global cancer statistics, HNSCC has high incidence and mortality rates worldwide, particularly in certain regions of Asia where smoking and alcohol consumption are prevalent (Barsouk et al., 2023; Michmerhuizen et al., 2016). Early symptoms of HNSCC are often subtle, leading to late-stage diagnoses in many patients, which not only complicates treatment but also significantly reduces patients’ quality of life and survival rates (Johnson et al., 2020). Therefore, investigating the pathogenesis of HNSCC and identifying effective early diagnostic markers and therapeutic targets are crucial for improving patient prognosis.

Currently, HNSCC treatment primarily involves surgery, radiotherapy, and chemotherapy (Qin et al., 2021; Bozec et al., 2019). However, due to the heterogeneity of HNSCC, there are significant differences in disease progression and treatment responses among patients (Licitra et al., 2016; Alhiyari et al., 2020). Additionally, advanced HNSCC patients often respond poorly to conventional treatments, with treatment-related side effects severely impacting their quality of life. In recent years, a deeper understanding of the molecular mechanisms underlying HNSCC has led to the increasing application of novel therapeutic strategies such as targeted therapy and immunotherapy in clinical settings, offering new treatment options for HNSCC patients (Kozakiewicz and Grzybowska-Szatkowska, 2018; Ghosh et al., 2022). Nevertheless, the efficacy of these novel therapies is often influenced by individual patient differences, and some patients may develop resistance (Wallington‐Beddoe et al., 2018). Therefore, formulating personalized treatment plans based on patients’ molecular characteristics remains a significant challenge in HNSCC treatment.

Recent studies have highlighted the significant role of exercise-related genes in cancer biology, revealing their potential impact on tumor progression and patient prognosis. Exercise-related genes are known to modulate various physiological pathways, including metabolism, immune response, and cellular stress, all of which are critical in cancer development (Chen et al., 2024a; Zhu et al., 2022; Idorn and Thor Straten, 2017; Chen et al., 2024b). Understanding the expression patterns and functions of these genes in HNSCC could provide valuable insights into novel therapeutic targets and prognostic markers. In the realm of cancer therapy, protein drugs have emerged as a promising class of therapeutics due to their high specificity and ability to target complex molecular interactions within the tumor microenvironment (Zhang Y. et al., 2023). Protein drugs, often derived from natural proteins or engineered for enhanced stability and efficacy, can precisely modulate key signaling pathways and cellular processes. They offer unique advantages over small-molecule drugs, including reduced off-target effects and the capacity to engage with targets that are traditionally considered “undruggable” by conventional pharmacological approaches. A pivotal aspect of protein drug development lies in the understanding of post-translational modifications (PTMs), which are chemical alterations that proteins undergo after synthesis. PTMs, such as phosphorylation, ubiquitination, and glycosylation, significantly influence protein function, localization, and stability (Pan and Chen, 2022). In the context of cancer, PTMs play a crucial role in regulating oncogenes and tumor suppressors, thereby affecting tumor progression and response to therapy (Shu et al., 2023). Pharmacological interventions targeting PTMs hold great promise in cancer treatment, as they can disrupt aberrant signaling pathways and restore normal cellular functions.

Bioinformatics is an interdisciplinary field that integrates biology, computer science, and information technology to analyze and interpret biomedical data (Chen B. et al., 2022; Huang J. et al., 2022; Lin et al., 2022). Its application in HNSCC research is becoming increasingly widespread. Through the analysis of large-scale genomics, transcriptomic, and proteomic data, researchers can identify molecular markers related to the occurrence, development, and prognosis of HNSCC, unveiling the molecular mechanisms of the disease. For instance, gene expression profiling can reveal specific gene expression patterns in HNSCC patients, providing a basis for molecular classification and prognosis assessment (Lee et al., 2018). Protein-protein interaction network analysis can identify key regulatory factors and signaling pathways in HNSCC (Kuang et al., 2016). Additionally, bioinformatics assists in the screening and validation of drug targets, supporting precision therapy for HNSCC.

The role of big data and bioinformatics in identifying and applying biomarkers is increasingly important, particularly in disease diagnosis and prognosis evaluation (Jiang et al., 2023). Continuous research into gene expression and regulatory mechanisms in disease studies has provided essential insights into disease onset and progression. Through multi-omics analyses and chemical proteomics studies, scientists have revealed the significance of gene regulatory networks in cellular function regulation (Qin et al., 2024). The utilization of big data and bioinformatics technologies in biomarker identification and application has become increasingly significant for disease diagnosis and prognosis evaluation. For instance, deep learning and multi-omics analyses enable scientists to more precisely identify and validate disease-related biomarkers (Liang et al., 2024; Xia et al., 2024). Research into gene expression and regulatory mechanisms has deepened, offering critical insights into disease onset and progression (Wang et al., 2024; Sun et al., 2024). Identifying gene signatures associated with specific diseases allows for comprehensive analyses, revealing stress responses related to cognitive impairment and aging (Lin et al., 2022). Single-cell RNA sequencing and bioinformatics analyses also help identify key molecules and pathways related to the tumor microenvironment, guiding precision therapy (Wang et al., 2024; Zhang et al., 2024).

Recent studies indicate that regulating specific biomolecules and applying certain compounds can address various biological responses (Du and Liu, 2024). Bioinformatics technology has been essential in studying gene expression and regulatory mechanisms, enhancing our understanding of biological processes (Du and Liu, 2024; Logan et al., 2024). Integrative research combing clinical and genomics data has developed various models and tools for predicting disease progression and treatment response, improving disease prediction accuracy and supporting personalized medicine (Figueredo, 2024; Li M. et al., 2024). Through deep learning and multi-omics analyses, scientists can more accurately identify and validate disease-related biomarkers (Scimeca et al., 2024). Research analyzing big data and bioinformatics has revealed associations between physical activity and cognitive function in older adults, providing new perspectives for healthy aging (Chen Y. et al., 2022). Studies on gene expression and regulatory mechanisms in diseases have deepened, using genome-wide association studies and polygenic risk scores to predict disease risk and shared phenotypes, offering critical insights into disease onset and progression (Yan et al., 2024). Multi-omics integrative analyses and bioinformatics methods allow researchers to comprehensively understand the multidimensional characteristics of diseases, advancing the development of personalized therapies (Chen B. et al., 2022). Genomics editing technologies have demonstrated significant potential in metabolic diseases, hormonal systems, and disease research, driving the development of precision medicine (Liu P. et al., 2023). The application of these advanced technologies and methodologies has not only propelled the development of biomedical research but also provided a solid foundation for the realization of precision medicine (Huang L. et al., 2022; Huang et al., 2013; Wang et al., 2005; Huang et al., 2015).

We will investigate the effects of changes in PIGF gene expression on the biological behaviors of HNSCC cells, including proliferation, migration, invasion, and apoptosis, through *in vitro* cellular experiments. Additionally, we will explore the relationship between PIGF gene expression and the tumor immune microenvironment, as well as the potential mechanisms of PIGF gene in HNSCC. Finally, we will evaluate the potential of PIGF as a therapeutic target for HNSCC, providing scientific evidence for the development of new therapeutic strategies.

Through this study, we aim to provide new insights into the early diagnosis, treatment strategy formulation, and prognosis evaluation of HNSCC. Simultaneously, our research will offer a new perspective on the relationship between exercise and HNSCC prognosis, providing a theoretical basis for developing exercise-based preventive and therapeutic strategies for HNSCC.

## 2 Materials and methods

### 2.1 Identification of exercise-related prognostic genes in HNSCC

In this study, we used a large bioinformatics approach to identify genes associated with exercise and survival prognosis in HNSCC. Transcriptome data for HNSCC were initially downloaded from The Cancer Genome Atlas (TCGA) database. Statistical analyses were conducted to identify genes exhibiting significant differential expression between HNSCC tissues and normal tissues. Subsequently, survival analysis was performed on these differently expressed genes to pinpoint those closely linked to patient prognosis. Gene sets related to exercise, encompassing various biological processes associated with exercise response, were obtained from the Gene Set Enrichment Analysis (GSEA) database. The intersection analysis on differentially expressed genes and exercise-related gene sets was conducted to obtain the key genes using public GSEA database. We also demonstrated that these genes were not only exercise-response related but also extremely correlated with the survival prognosis of HNSCC patients. The identification of these resplices provides new insights into the molecular biology underlying HNSCC and may yield novel.

### 2.2 Functional analysis of exercise-survival prognosis-related genes in HNSCC

Gene Ontology (GO) and Kyoto Encyclopedia of Genes and Genomes (KEGG) enrichment analyses were conducted on the selected genes to elucidate their distribution in biological processes, molecular functions, and cellular components, as well as their involvement in specific metabolic and signaling pathways. Gene Set Enrichment Analysis (GSEA) was then employed to investigate the expression patterns of these genes under various biological conditions, aiming to identify gene sets that may influence the survival prognosis of HNSCC patients. And survival prognosis analysis was conducted to test relationship between these gene expression levels and overall survival (OS) rate with disease free survival rate. Single-gene expression analysis was also conducted to delve into the expression patterns of each gene and their associations with the clinical characteristics and prognosis of HNSCC patients. These identification results are helpful in deepening understanding of the molecular mechanism for HNSCC and lay a scientific foundation in exploring novel therapeutic targets and prognostic biomarkers.

### 2.3 Pan-cancer expression landscape analysis of core genes

In the study of HNSCC, tumor samples were divided into two groups based on high and low PIGF expression levels. Differential expression analysis was conducted using the limma package, identifying significantly differently expressed genes, which were visualized with volcano plots. Protein-protein interaction data were filtered through the ComPPI database to exclude biologically unreasonable interactions, and interaction scores were introduced to quantify data accuracy. ROC analysis was performed using the pROC package to calculate the 95% confidence interval and AUC, and ROC curves were plotted to evaluate the diagnostic efficiency of gene expression in distinguishing tumor from normal tissues. The data were sourced from TCGA-corrected RNA-seq data, generated through the Firehose pipeline and normalized. Z-score standardization was used to identify outliers, and the Wilcoxon rank-sum test assessed expression differences between tumor and normal tissues. Furthermore, gene expression was normalized using Z-score after pairing data from the GTEx database with TCGA data to eliminate outliers, followed by ROC analysis to evaluate diagnostic performance. The Wilcoxon rank-sum test was used to compare PIGF expression between tumor and normal tissues. Calibration curves and goodness-of-fit tests were applied to assess the predictive accuracy of the models. GEO datasets were processed by converting probe matrices to gene matrices and applying Z-score standardization with the Wilcoxon rank-sum test evaluating expression differences between tumor and normal tissues. Additionally, six molecular immune subtypes associated with tumor characteristics and prognosis were evaluated using median grouping and chi-square tests to assess the significance of subtype proportions. The Kruskal-Wallis rank-sum test compared PIGF expression across different molecular subtypes, while clinical variables were statistically analyzed in different expression groups using median grouping and chi-square tests.

### 2.4 Prognostic analysis of core gene survival

In tumor tissues, the Pearson correlation between the target gene and both mRNA and miRNA was calculated, with scatter plots used to display these relationships. Results were reported only when the absolute value of the correlation coefficient exceeded 0.3. Gene expression levels were categorized based on their correlation strength with the target gene into four classes: strongly positive, moderately positive, weakly positive, and negative correlations. These were visualized using a heatmap of contingency tables, and Fisher’s exact test was employed for statistical analysis. Kaplan-Meier survival analysis was used to evaluate the correlation between gene expression levels and patient survival times. Detailed survival data analysis was performed using the survival package in R. The survminer package was used to identify optimal cut-off values for high and low expression groups, ensuring that the sample sizes for these groups met statistical requirements, typically not less than 30% of the total sample size. Moreover, a meta-analysis that based on univariate Cox proportional hazards model was performed by using the inverse variance method. We chose the hazard ratios (HR) as our primary measure of effect size, separating potential tumor-suppressive versus oncogenic actions. This simple classification approach does not consider the biological aspects of these genes. The statistical analysis and visualization were performed in R (version 4.3.2) using the meta package, which provides a range of functions for conducting meta-analyses and generating forest and funnel plots, visually presenting the combined effect sizes and assessing publication bias.

### 2.5 Core gene GSEA/GSVA enrichment analysis

The study had used a stratified approach to classify samples as high or low expression group basing on the top 30% of most expressed samples versus bottom 30%. This second classification aimed to find the most extreme gene expression changes associated with disease states. This was followed up by a differential expression analysis using the limma package, producing log2 fold changes (log2FC) and ranking genes that were statistically altered. The additional analysis utilizes gene sets from the KEGG database and was performed with fgsea function in the R package fgsea. Enrichment scores (ES) of gene sets with significant P values were calculated by using GSEA analysis, and tested for significance/multiple hypothesis correction was applied. Gene Sets with uncorrected p-value < 0.05 and corrected p-value < 0.25 were assumed to have biological significance and visualized as before based on species partitioning. To explore the states of tumor cells better in single-cell level, we performed CancerSEA analysis. This platform creates a merged view of the datasets and uncovers 14 new functional states that are able to articulate significant aspects of tumor cell function, making it usable as an effective resource for conducting meaningful experiments. Using the z-score algorithm proposed by Lee et al., gene set values were calculated and converted to z-scores with the GSVA algorithm in the R package GSVA. Pearson correlation analysis was then employed to explore the relationships between gene expression and functional states, calculating the correlation between gene expression and gene set z-scores. Finally, the gsva function in the GSVA package was used to score 73 metabolic gene sets from the KEGG database. Based on these GSVA scores, the limma package was again used to compare metabolic pathway activities between the high and low expression groups, revealing the role of metabolic pathways in disease progression.

### 2.6 Sensitivity of core genes to immunotherapy

Gene expression data from multiple publicly available datasets of cancer patients undergoing immunotherapy were utilized. To assess the diagnostic performance of PIGF expression in distinguishing between responders and non-responders to immunotherapy, ROC curve analysis was conducted using the pROC package in R. The area under the curve (AUC) and 95% confidence intervals (CI) were calculated, and smoothing techniques were applied to the ROC curves for improved visualization. Patients were categorized into high and low PIGF expression groups based on the median expression level of PIGF. A Chi-square test was performed to examine the differences in the proportions of responders and non-responders between these two groups. Furthermore, the Wilcoxon rank-sum test was employed to compare gene expression differences between responders and non-responders, and gene expression levels were standardized into Z-scores for statistical comparison.

### 2.7 Immune infiltration analysis of core genes

The TIMER 2.0 database was employed to collect and analyze immune infiltration data from TCThe TIMER 2.0 database was used in this study to obtain immune infiltration data from the TCGA tumor samples5. We developed a comprehensive immune-related long non-coding RNAs database and discovered that multiple algorithms can evaluate the abundance of different types of immune cells in tumor tissues, and analyze their relationship with gene expression. This approach served data quality and provenance, giving a holistic overview on the interaction of immune cells to gene expression. The bar of scatter plots shows us correlation coefficient, which clearly describes the relationship between immune cell type and gene expression. Samples were bimodalized based on median of gene expression as an robust estimator to differentiate between low and hight expressions people — the healthy group in blue line while ICH is presented by red solid line. To find whether the immune cell contents in high and low expression groups had statistical differences, Wilcoxon rank sum test was performed as a nonparametric method applied to comparisons among different kinds of data distributions. Heatmaps were generated to show key immune cell types in detail.

### 2.8 Mutation analysis of core genes

Whole-genome CRISPR screening data were obtained from the DepMap portal, and dependency scores for approximately 17,000 candidate genes were analyzed using the CERES algorithm. The pan-cancer mutation landscape of core genes was visualized using the plotmafSummary function from the maftools package. To assess the independence between gene expression levels and specific gene mutation types the independence_test function from the R coin package, based on permutation tests, was employed. Genes with a mutation rate exceeding 10% and a p-value less than 0.01 were identified and visualized to highlight significant associations between gene expression and mutation types. In the TCGA-HNSC project, copy number variation (CNV) analysis was performed using the GISTIC score method to identify genomics CNVs. The CNV profile of 451 samples was visualized using bar plots, which reflected chromosomal copy number changes. Quantitative measures of genomics alterations, such as FGA, FGG, and FGL, were defined based on the genomics distance of clonal regions. When analyzing differences between specific gene expression subgroups, ANOVA was used, followed by Tukey’s Honest Significant Difference (TukeyHSD) test for multiple comparisons if ANOVA indicated significance. This approach was used to identify specific group differences. The correlation between CNV scores and gene expression levels was analyzed using scatter plots combined with Spearman’s rank correlation coefficient, which measures the monotonic relationship between two variables. CNV data were obtained from the TCGA Genome Characterization Center andmeasured through whole-genome arrays. Gene-level copy number estimates were derived using the TCGA FIREHOSE pipeline and GISTIC2 method. The Kruskal-Wallis test, a non-parametric method for comparing multiple samples, was employed to compare gene expression differences among different CNV types (ranging from −2 to 2).

### 2.9 Single-gene pan-cancer single-cell sequencing analysis

Single-cell gene expression data for HNSCC were obtained from the TISCH database. Heatmaps, generated using the pheatmap package, effectively revealed gene expression patterns at the single-cell level across different cancer types. Hierarchical clustering analysis, performed using Euclidean distance and Ward’s minimum variance method, uncovered intrinsic patterns of gene expression and their conservation across various cancers. Additionally, UMAP (Uniform Manifold Approximation and Projection) was utilized to explore expression patterns in high-dimensional data, preserving the original data topology during dimensionality reduction. By using UMAP analysis of gene expression data for CENPF, we could render a clearer depiction of the patterns underlying adding new observations to key biological discovery. To determine significant differences in specific gene expression between cell types, the Kruskal-Wallis rank sum (KW) test was applied as a non-parametric method suitable for un-normally distributed samples. Furthermore, UMAP visualization of the AUC cell scoring capturing heterogeneity of pathway activity in individual cells was performed. Methodology for spatial transcriptomics can be found in the supplementary methods.

### 2.10 Cell proliferation assay

Cell proliferation was determined by the cell counting Kit-8 (CCK-8, Beyotime Biotechnology Co., Ltd., Shanghai, China) at 0 h, 24 h and 48 through various treatments. The cells were then cultured in 96-well plates (Thermo Fisher Scientific. MA, United States) and exposed to respective interventions for another 24 h essentially as described above briefly After that, 10 μL of CCK-8 solution was added to each well and incubated for another 2 h. Absorbance (450 nm) was determined by a Microplate Reader (Bio-Rad, Hercules, CA). Cell viability was evaluated by calculating the ratio of the average absorbance of the treated groups to that of the control group, expressed as a percentage ([Absorbance of treated group/Absorbance of control group] × 100%).

### 2.11 Clonogenic assay for cell proliferation

Cells in the logarithmic growth phase were collected and diluted to a concentration of 500 cells/mL. Each well of a 6-well plate was pre-wetted with 1 mL of culture medium before adding 1 mL of the cell suspension. Three replicate wells were prepared for each group. The cells were incubated overnight in a 37°C, 5% CO_2_ incubator to allow for attachment. Subsequently, cells were collected and 5 × 10^4 cells per well were added to the corresponding wells, with the medium being changed every 2 days. After 12 days, the medium was discarded, and the wells were washed twice with PBS. Cells were then fixed by adding 1 mL of methanol to each well and incubating at room temperature for 20 min. After removing the methanol, 1 mL of 0.1% crystal violet was added for staining at room temperature for 20 min. The wells were then washed with PBS until the background was clear, followed by photographing and counting the colonies.

### 2.12 Transwell and scratch assays for assessing cell invasion and migration

SCC4 cells were seeded into 6-well plates at a density of 5 × 10^5^ cells per well. After 12 h of incubation, a sterile pipette tip was used to create scratches along predefined tracks. Detached cells were washed away with PBS, and photographs were taken. The plates were then returned to the incubator for an additional 24 h, followed by another round of photography to capture cell migration. For the Transwell assay, treated SCC4 cells were seeded into the upper chamber of Transwell inserts pre-coated with Matrigel at a density of 1 × 10^5^ cells per well. The lower chamber was filled with 600 μL of culture medium containing 5% fetal bovine serum. The cells were incubated for 24 h, after which they were fixed with 4% paraformaldehyde for 20 min and stained with 0.1% crystal violet for 10 min. The number of invading cells was then observed under a microscope and photographed.

### 2.13 Statistical analysis

The statistical analysis was conducted using SPSS software version 26.0 (SPSS Inc., Chicago, United States). The results are expressed as the mean ± standard deviation. For comparisons between two groups, Student’s t-test was utilized, while one-way ANOVA was applied for comparisons across multiple groups. Statistical significance was defined as P-value < 0.05.

## 3 Results

### 3.1 Identification and multigene analysis of exercise-survival prognosis-related genes in HNSCC

**FIGURE 1:**
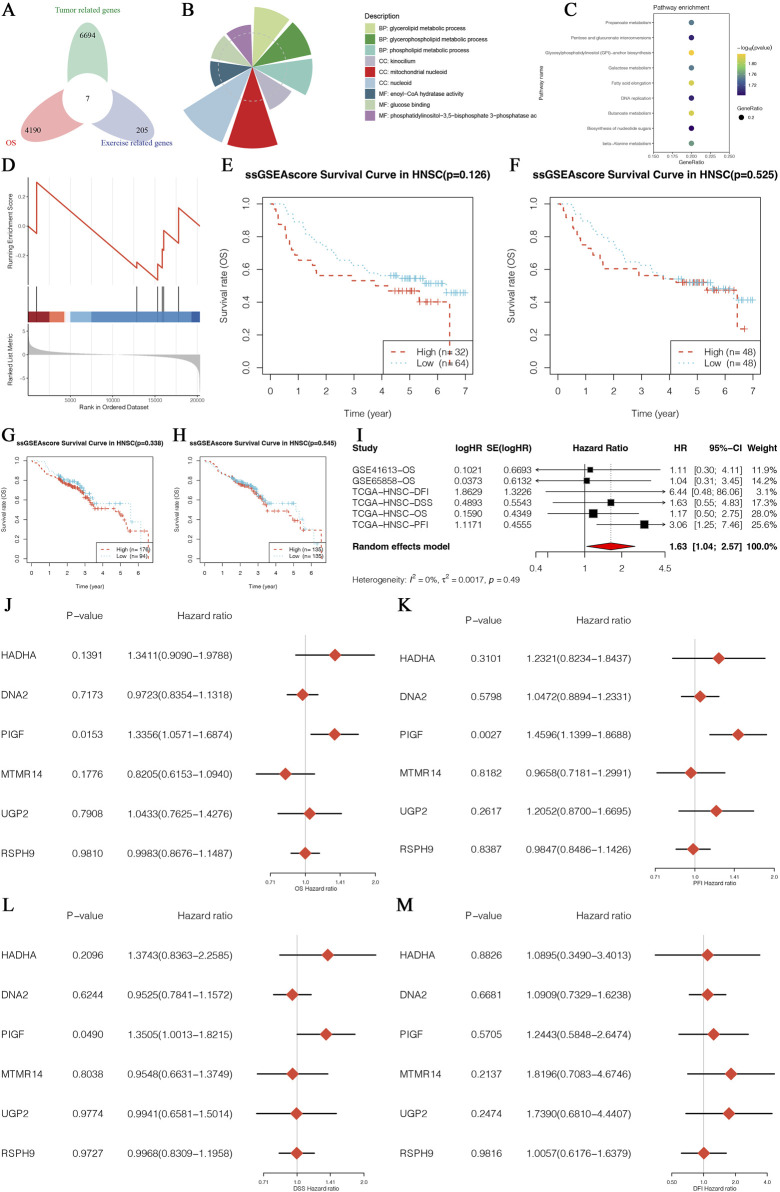
Identification and Multigene Analysis of Exercise-Survival Prognosis-Related Genes in HNSCC. **(A)** Venn diagram showing the intersection of exercise-related genes, tumor-related genes, and survival prognosis-related genes in HNSCC. **(B)** Pie chart depicting the distribution of core genes enriched in various biological processes and pathways based on Gene Ontology (GO) analysis. **(C)** Dot plot displaying Kyoto Encyclopedia of Genes and Genomes (KEGG) pathway enrichment analysis for the core genes, highlighting significant pathways involved in HNSCC. **(D)** Enrichment plot from Gene Set Enrichment Analysis (GSEA) indicating the enrichment of core genes in specific biological pathways or processes. **(E)** Survival curve based on the single-sample Gene Set Enrichment Analysis (ssGSEA) score for a core gene in the HNSCC patient cohort from GSE126 datasets. **(F)** Survival curve based on the ssGSEA score for a core gene in the HNSCC patient cohort from the GSE525 datasets. **(G)** Kaplan-Meier survival analysis for a core gene in the HNSCC patient cohort from the GSE407 datasets. **(H)** Kaplan-Meier survival analysis for a core gene in the HNSCC patient cohort from the GSE123 datasets. **(I)** Forest plot of hazard ratios from a meta-analysis of survival data, showing the combined effect size and confidence intervals for core genes across multiple datasets. **(J–M)** Forest plots of hazard ratios from multivariate Cox regression analysis for multiple core genes, illustrating their independent prognostic value in HNSCC.

The results of this study present the identification and multigene analysis of exercise-survival prognosis-related genes in HNSCC, as illustrated in Figure 1. The Venn diagram (Figure 1A) highlights the intersection of exercise-related genes, tumor-related genes, and survival prognosis-related genes in HNSCC, pinpointing core genes crucial for survival prognosis influenced by exercise. The distribution of these core genes across various biological processes and pathways is depicted in the GO analysis pie chart (Figure 1B), emphasizing their involvement in diverse cellular functions. Further insights are provided by the KEGG pathway enrichment analysis (Figure 1C), which identifies significant pathways involving these core genes. The GSEA plot (Figure 1D) demonstrates the enrichment of core genes in specific biological pathways or processes, indicating their functional relevance in the context of HNSCC and exercise. Survival analyses using the ssGSEA score are presented for different HNSCC patient cohorts (Figures 1E, F, datasets GSE126 and GSE525), revealing the association between core gene expression levels and overall survival. Additional Kaplan-Meier survival analyses (Figures 1G, H, datasets GSE407 and GSE123) consistently show the correlation between core gene expression and patient survival, reinforcing their prognostic value. A meta-analysis of survival data (Figure 1I) confirms the significant prognostic impact of these genes across multiple datasets. Multivariate Cox regression analyses (Figures 1J–M) further illustrate the independent prognostic value of these genes, highlighting their potential as biomarkers for HNSCC prognosis. These comprehensive analyses demonstrate the critical role of exercise-survival prognosis-related genes in HNSCC and their potential as prognostic biomarkers, providing valuable insights into the molecular mechanisms of HNSCC influenced by exercise.

### 3.2 Expression of PIGF in HNSCC using TCGA data

**FIGURE 2:**
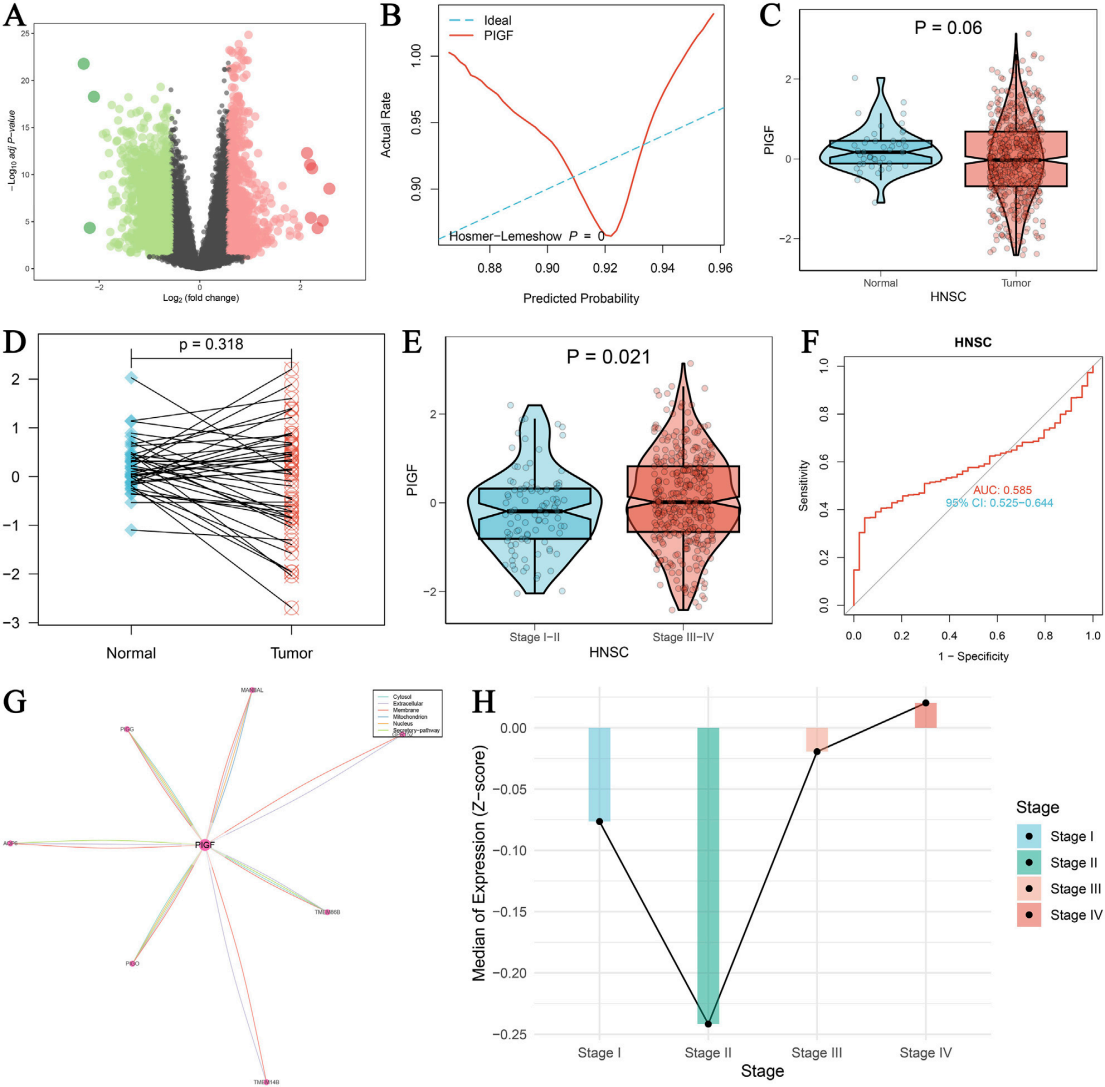
Expression of PIGF in HNSCC using TCGA data. **(A)** Volcano plot visualizing the differential expression of PIGF using the limma package. The log2 fold change is plotted against the -log10 adjusted p-values. Significant upregulation of PIGF is indicated by red dots, while significant downregulation is indicated by green dots. **(B)** ROC curve evaluating the diagnostic performance of PIGF expression in distinguishing tumor tissues from normal tissues. The Hosmer-Lemeshow goodness-of-fit test is included, with the predicted probability plotted against the actual rate. The curve reflects the ability of PIGF to predict tumor presence, with an AUC value provided. **(C)** Violin plot comparing the expression levels of PIGF between normal and tumor tissues in HNSCC. Statistical significance is indicated (p = 0.06). **(D)** Paired sample plot showing the expression of PIGF in matched normal and tumor tissues, with a p-value indicating the significance of the difference. **(E)** Violin plot depicting the expression of PIGF in HNSCC across different tumor stages (Stage I-II vs. Stage III-IV). Statistical significance is indicated (p = 0.021). **(F)** ROC curve assessing the diagnostic performance of PIGF expression in differentiating early-stage (Stage I-II) from advanced-stage (Stage III-IV) HNSCC. The AUC value and confidence interval (CI) are provided. **(G)** Interaction analysis of PIGF with other genes, visualized as a network. The central node represents PIGF, with edges indicating interactions with other genes. The thickness of the edges correlates with the strength of interaction. **(H)** Median expression levels of PIGF in HNSCC across different tumor stages (Stage I, II, III, IV), represented by the Z-score of expression values. Each bar indicates the median expression level for the corresponding stage, with significant differences highlighted.

The study focused on analyzing the expression pattern of the PIGF gene in HNSCC using data from The Cancer Genome Atlas (TCGA). Figure 2A displays a volcano plot generated through differential expression analysis. The diagnostic potential of PIGF in distinguishing tumor tissues from normal tissues is evaluated by the ROC curve in Figure 2B, which presents an AUC value indicating PIGF’s predictive ability. Further analysis in Figure 2C shows a violin plot comparing PIGF expression levels between normal and tumor tissues, approaching statistical significance (p = 0.06), while Figure 2D presents a paired sample plot indicating a non-significant difference (p = 0.318). The expression pattern across different tumor stages is shown in Figure 2E, with significant differences (p = 0.021) between early-stage (Stage I-II) and advanced-stage (Stage III-IV) HNSCC. Figure 2F’s ROC curve assesses the diagnostic performance of PIGF in differentiating early from advanced stages, demonstrating moderate efficacy. Figure 2G visualizes the gene interaction network with PIGF as the central node, indicating its interactions with other genes. Finally, Figure 2H shows the median expression levels of PIGF at different tumor stages, emphasizing the dynamic changes in expression during tumor progression. The expression level of PIGF was significantly upregulated in stage III and IV HNSCC, showing a linear relationship, indicating a correlation with disease severity. However, the expression level of PIGF in stage II HNSCC is lower than that in stage I. These results collectively underscore the significant upregulation of PIGF in HNSCC, its diagnostic potential, and its variable expression across different tumor stages, enhancing our understanding of its role in cancer progression.

### 3.3 Multidimensional analysis of PIGF gene in HNSCC

**FIGURE 3:**
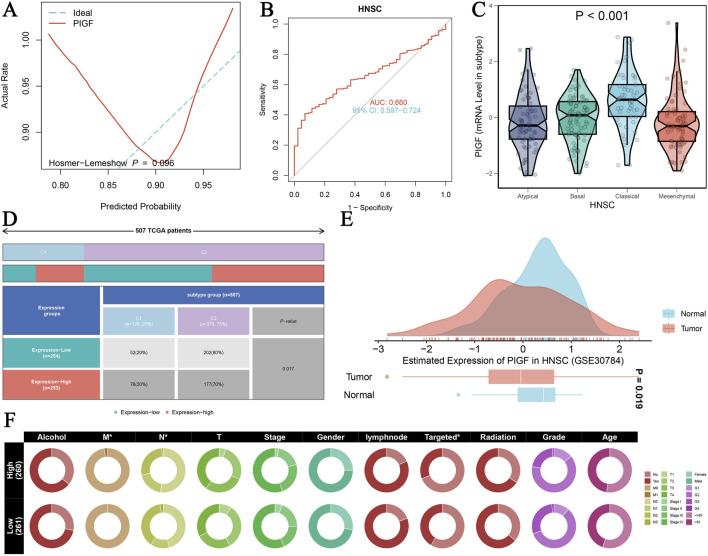
PIGF in HNSCC Analyzed with TCGA-GTEX Data. **(A)** ROC curve assessing the diagnostic efficiency of PIGF expression between tumor and normal groups. **(B)** ROC curve evaluating the diagnostic performance of PIGF expression distinguishing tumor from normal tissue in HNSCC. **(C)** Differential expression of PIGF gene across various tumor subtypes of HNSC. The violin plot displays the distribution of PIGF mRNA levels in atypical, basal, classical, and mesenchymal subtypes with a significant p-value <0.001. **(D)** Heatmap illustrating the expression difference of the PIGF gene in HNSCC from TCGA patients. The data include expression levels categorized by high and low expression groups, along with the corresponding patient subtypes. **(E)** Density plot depicting the estimated expression of PIGF in HNSCC using data from GSE30784. The plot compares the expression levels between normal and tumor tissues, highlighting significant differences. **(F)** Chi-square test results showing the association between PIGF expression levels and various clinical traits in HNSCC patients.

A comprehensive analysis of the PIGF gene in HNSCC using TCGA-GTEX data provides significant insights into its diagnostic and clinical relevance (Figures 3A–F). Figure 3A shows the ROC curve assessing the diagnostic efficiency of PIGF expression between tumor and normal groups, including the Hosmer-Lemeshow test with a p-value of 0.096, indicating a good model fit. Figure 3B further highlights the diagnostic performance of PIGF expression in distinguishing tumor from normal tissue in HNSCC, with an area under the curve (AUC) of 0.660 and a 95% confidence interval of 0.597–0.724, demonstrating high diagnostic accuracy. The differential expression of the PIGF gene across various tumor subtypes of HNSCC is depicted in Figure 3C. The violin plot illustrates the distribution of PIGF mRNA levels in atypical, basal, classical, and mesenchymal subtypes, with a significant p-value of less than 0.001, indicating notable differences among subtypes. Figure 3D presents a heatmap showing expression differences of the PIGF gene in HNSCC patients from TCGA, categorizing expression levels into high and low groups and correlating these with patient subtypes. Figure 3E shows a density map comparing the estimated expression of PIGF, showing the obvious differences between normal and tumor tissues. The results showed that the expression level of PIGF in tumor tissues was significantly higher than that in normal tissues. Chi square test results (Figure 3F) highlighted the association between PIGF expression level and various clinical characteristics of hNSC patients, including alcohol consumption, HPV status, lymph node involvement, tumor stage, gender, targeted therapy, radiotherapy, tumor grade and patient age, discriminated between high expression group and low expression group and provided the relevant p value. The results showed that patients with high PIGF expression had later tumor stage, more lymph node involvement, and were more sensitive to targeted therapy and radiotherapy. These findings collectively reveal the multidimensional characteristics of the PIGF gene in HNSCC, underscoring its potential as a diagnostic biomarker and its association with clinical traits.

### 3.4 Analysis of PIGF gene Interactions

**FIGURE 4:**
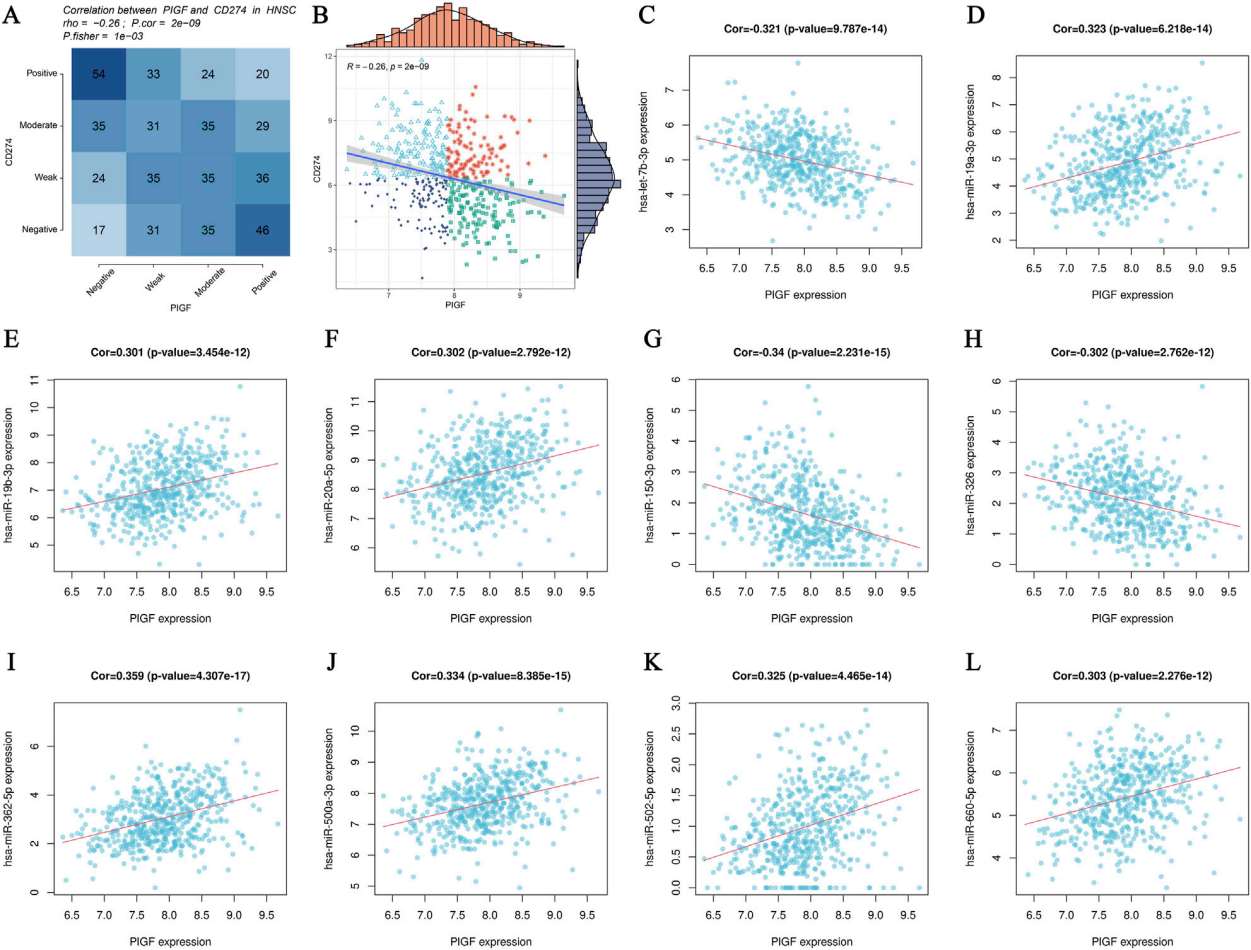
Analysis of PIGF Gene Interactions. **(A, B)** Pearson correlation analysis scatter plots showing the relationship between PIGF mRNA expression and various related mRNAs. **(A)** Heatmap displaying the correlation matrix between PIGF mRNA expression and other mRNAs. The color intensity represents the strength of the correlation, with blue indicating negative correlation and red indicating positive correlation. **(B)** Scatter plot with a regression line depicting the Pearson correlation between PIGF mRNA expression and a specific related mRNA. The *x*-axis represents the expression levels of PIGF mRNA, while the *y*-axis represents the expression levels of the related mRNA. The distribution of data points and the regression line illustrate the direction and strength of the correlation. **(C–L)** Pearson correlation analysis scatter plots showing the relationship between PIGF mRNA expression and various related miRNAs. Each plot represents the correlation of PIGF mRNA with a different miRNA.

Pearson correlation analysis is used to identify significant interactions between *PIGF* expression and various related mRNAs and miRNAs. Figure 4A presents a heatmap displaying the correlation matrix between *PIGF* mRNA and other mRNAs, with color intensity indicating the correlation strength—blue for negative and red for positive correlations. Figure 4B features a scatter plot with a regression line, illustrating the Pearson correlation between *PIGF* mRNA expression and a specific related mRNA. The *x*-axis represents *PIGF* mRNA levels, and the *y*-axis represents the related mRNA levels, highlighting the direction and strength of the correlation. Furthermore, Figures 4C–L depict scatter plots showing the correlation between PIGF mRNA and various miRNAs. Each plot, such as Figure 4C, includes the correlation coefficient and p-value, indicating a statistically significant positive correlation. The analysis reveals moderate to strong correlations between PIGF mRNA and these mRNAs and miRNAs, with significant p-values. These findings suggest that PIGF may play a crucial role in the regulatory network involving these genes, providing a foundation for future studies on the functional implications of PIGF interactions in the studied condition.

### 3.5 PIGF gene prognostic survival analysis

**FIGURE 5:**
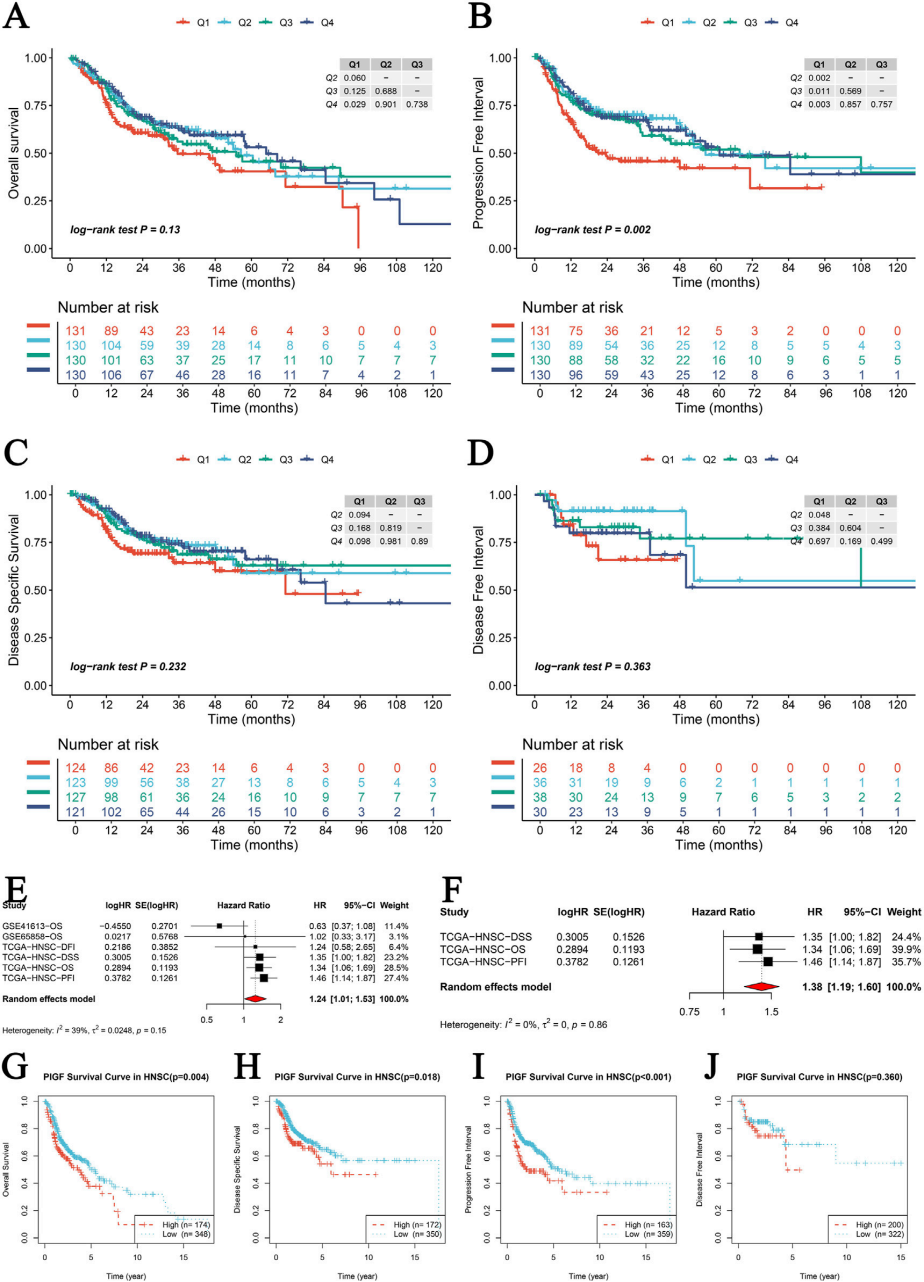
PIGF Gene Prognostic Survival Analysis. **(A–D)** Kaplan-Meier survival analysis for four survival metrics including Overall Survival (OS), Disease-Specific Survival (DSS), Progression-Free Interval (PFI), and Disease-Free Interval (DFI). Each plot represents the survival curves for different quartiles (Q1-Q4) of PIGF expression levels, with Q1 representing high expression of PIGF and Q4 representing low expression of PIGF. Log-rank test p-values are provided to show the statistical significance of differences between the curves. **(A)** Overall Survival (OS): Log-rank test P = 0.13. **(B)** Progression-Free Interval (PFI): Log-rank test P = 0.002. **(C)** Disease-Specific Survival (DSS): Log-rank test P = 0.232. **(D)** Disease-Free Interval (DFI): Log-rank test P = 0.363. **(E, F)** Meta-nalysis of univariate Cox regression survival analysis across multiple datasets. Forest plots showing the hazard ratios (HR) and 95% CI for PIGF expression in different studies. **(E)** Univariate Cox regression analysis combining multiple datasets. The pooled HR indicates the overall effect of PIGF expression on survival. **(F)** Hazard ratios from individual studies in the TCGA datasets for different survival outcomes. The random effects model is used, and heterogeneity statistics are provided. **(G–J)** External GEO datasets survival prognostic analysis of PIGF expression. **(G)** Kaplan-Meier survival curve in HNSCC (GSE10406) with a p-value of 0.004. **(H)** Kaplan-Meier survival curve in HNSCC (GSE84318) with a p-value of 0.016. **(I)** Kaplan-Meier survival curve in HNSCC (GSE53161) with a p-value of 0.001. **(J)** Kaplan-Meier survival curve in HNSCC (GSE24362) with a p-value of 0.348.

A comprehensive analysis is conducted to evaluate the prognostic significance of PIGF gene expression across various survival outcomes using internal and external datasets. Kaplan-Meier survival analysis is performed for four key survival outcomes: Overall Survival (OS), Disease-Specific Survival (DSS), Progression-Free Interval (PFI), and Disease-Free Interval (DFI). Survival curves are stratified by quartiles (Q1-Q4) of PIGF expression levels (Figures 5A–D). The results indicate no significant difference in OS (log-rank test P = 0.13; Figure 5A), a significant difference in PFI (log-rank test P = 0.002; Figure 5B), no significant difference in DSS (log-rank test P = 0.232; Figure 5C), and no significant association with DFI (log-rank test P = 0.363; Figure 5D). A meta-analysis of univariate Cox regression survival analysis across multiple datasets determines that higher PIGF expression correlates with an increased risk of poor survival outcomes (Figures 5E, F). External GEO datasets analysis validates these findings in head and neck squamous cell carcinoma (HNSCC), with significant associations observed in datasets GSE10406 (P = 0.004; Figure 5G), GSE84318 (P = 0.016; Figure 5H), and GSE53161 (P = 0.001; Figure 5I), though no significant association is found in GSE24362 (P = 0.348; Figure 5J). Further independent prognostic analysis confirmed the significance of PIGF expression independent of clinical variables through both univariate and multivariate Cox regression analyses (Supplementary Figures 1A–D). Expression levels are correlated with overall survival status (Supplementary Figure 1E), and a Chi-square test indicated no significant distribution difference across expression quartiles (P = 0.706; Supplementary Figure 1F). Restricted cubic spline models explored non-linear risk associations adjusted for relevant covariates, showing estimated log hazard ratios with 95% confidence bands (Supplementary Figures 1G–J). Kaplan-Meier survival curves stratified by expression levels reveal significant differences, particularly for OS and DSS (Supplementary Figures 1K–N). This analysis underscores the significant prognostic value of PIGF gene expression for various survival outcomes, suggesting its potential as a biomarker for cancer prognosis.

### 3.6 Core gene GSEA/GSVA enrichment analysis in HNSCC

**FIGURE 6:**
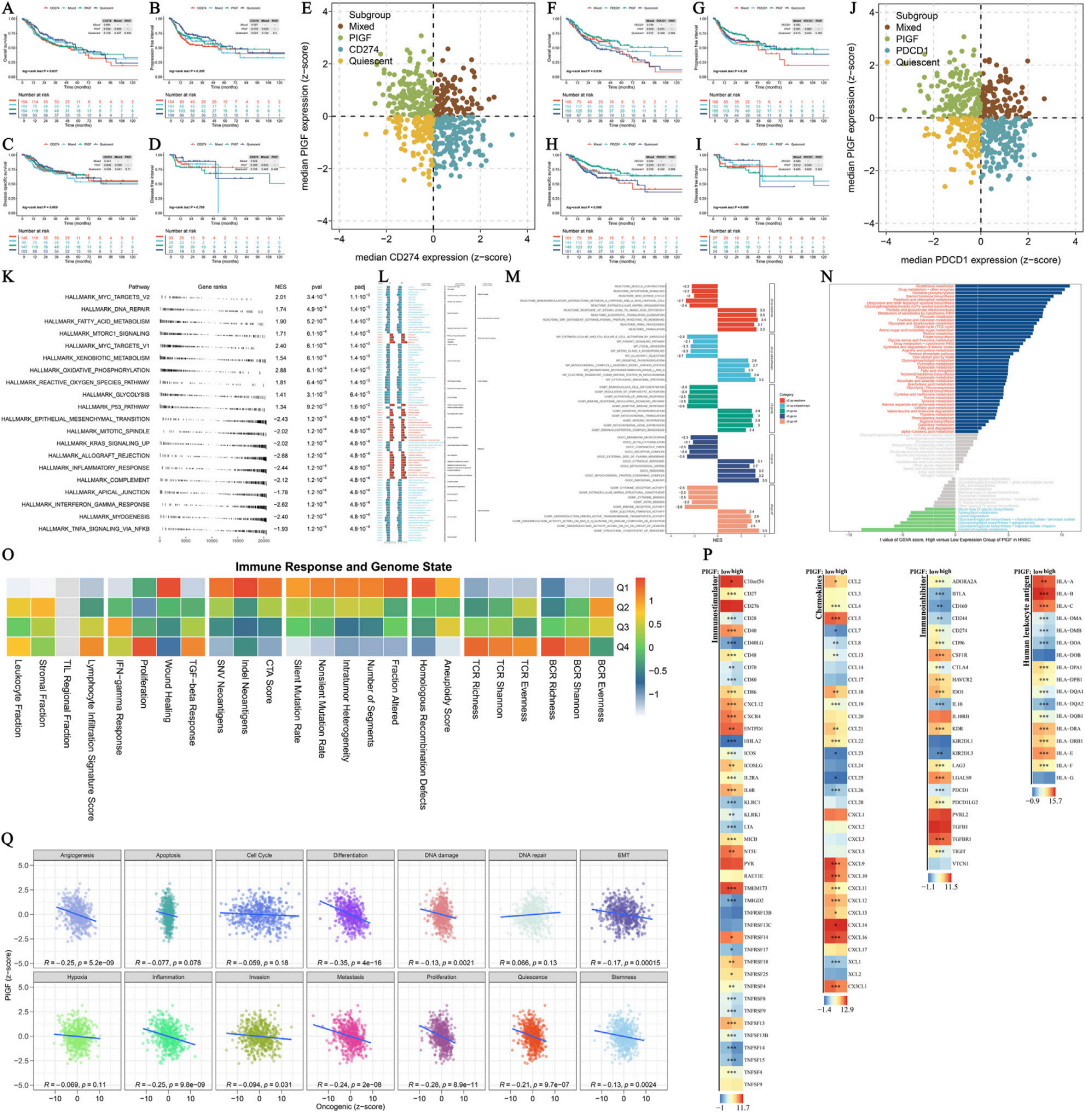
Core Gene GSEA/GSVA Enrichment Analysis. **(A–E)** Kaplan-Meier (KM) survival analysis for the four subgroups of dual-gene molecular subtype based on CD274 expression. Each subplot represents different survival curves comparing high and low expression groups of CD274, with statistical significance evaluated using log-rank tests. **(F–J)** Kaplan-Meier (KM) survival analysis for the four subgroups of dual-gene molecular subtype based on PDCD1 expression. Each subplot represents different survival curves comparing high and low expression groups of PDCD1, with statistical significance evaluated using log-rank tests. **(K)** GSEA for hallmark gene sets. This analysis compares the enrichment scores of high expression vs. low expression groups, highlighting significant pathways. **(L)** KEGG gene set enrichment analysis compares the enrichment scores between high and low expression groups, identifying key signaling pathways involved. **(M)** Multiple gene set enrichment analysis performed using the clusterProfiler package. The comparison is between high expression group to low expression group across various gene sets. The bar plot shows the normalized enrichment scores (NES) for significantly enriched pathways. **(N)** Differential GSVA scores for metabolic pathways between high and low expression groups of the core gene. The bar plot displays pathways with significant differences in GSVA scores, indicating altered metabolic activities. **(O)** Immune Response and Genome State heatmap. This heatmap illustrates the correlation between immune response signatures and genome state across different samples, indicating the interplay between immune activity and genetic alterations. **(P)** Landscape of PIGF in Immunostimulator. Heatmap representation of PIGF expression across various immune stimulators, highlighting its role in modulating immune responses. **(Q)** Pearson correlation analysis between z-scores of core gene expression and z-scores of 14 tumor state parameters. Scatter plots depict the correlation between core gene expression and various tumor-related parameters, with the Pearson correlation coefficient (r) and statistical significance (p-value) annotated.

The analysis of core genes in HNSCC provides comprehensive insights into their roles in tumor progression and patient survival outcomes through various Kaplan-Meier (KM) survival analyses, GSEA, and GSVA. Figures 6A–E demonstrate KM survival analyses for the four subgroups of dual-gene molecular subtypes based on CD274 expression. These figures show that high CD274 expression correlates with poorer survival outcomes. Similarly, Figures 6F–J demonstrate that elevated PDCD1 expression is associated with reduced survival rates. Figure 6K highlights significant pathways identified through GSEA for hallmark gene sets, revealing differently regulated biological processes. The KEGG gene set enrichment analysis in Figure 6L identifies critical signaling pathways involved in HNSCC, including those related to immune response and apoptosis. Figure 6M uses the clusterProfiler package to compare high and low expression groups, showcasing normalized enrichment scores (NES) for significantly enriched pathways, thereby emphasizing the diverse biological functions influenced by core genes. Differential GSVA scores for metabolic pathways in Figure 6N indicate altered metabolic activities linked to core gene expression. The heatmap in Figure 6O illustrates the correlation between immune response signatures and genome state, highlighting the interplay between immune activity and genetic alterations. Figure 6P represents PIGF expression across various immune stimulators, underscoring its role in modulating immune responses. Finally, Figure 6Q presents Pearson correlation analyses between z-scores of core gene expression and tumor state parameters, offering insights into the relationships between core gene expression and tumor-related parameters. This comprehensive analysis provides a detailed perspective on the functional roles of core genes in HNSCC, emphasizing their potential as prognostic biomarkers and therapeutic targets.

### 3.7 Single-cell sequencing analysis of PIGF in HNSCC

**FIGURE 7:**
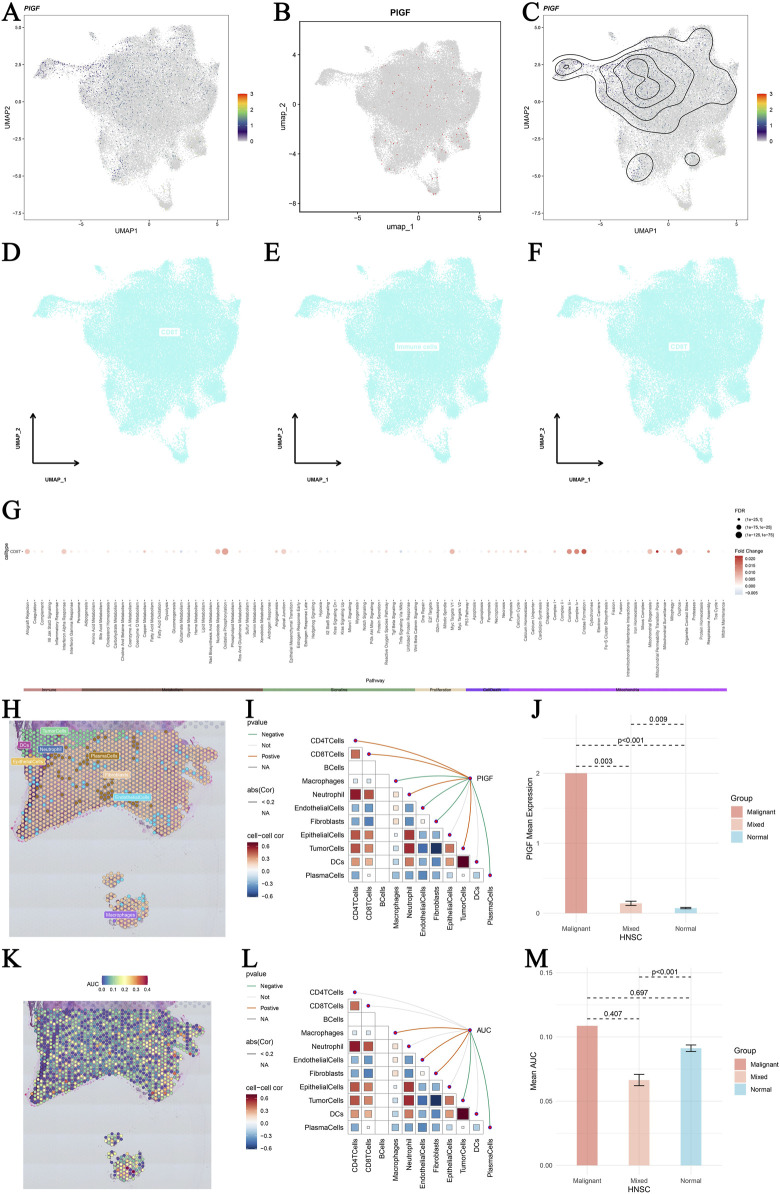
Single-cell sequencing analysis of PIGF in head and neck squamous cell carcinoma (HNSCC). **(A–C)** UMAP plots displaying major cell lineages **(A)** and single-cell PIGF gene expression **(B)** with density contour lines **(C)** in HNSCC single cells. The color gradient represents the expression levels of PIGF, with higher expression shown in darker colors. **(D–F)** UMAP plots showing differential expression of core genes across different cell types in HNSCC. Cells are colored based on core gene expression levels, highlighting the variability in gene expression among different cell populations. **(G)** Pathway differences between core gene-positive and core gene-negative groups across various cell types. Dot plot representing the pathways enriched in each cell type, with the size of the dots indicating the number of genes involved and the color gradient representing the significance of the enrichment (p-values). **(H)** Spatial transcriptomic deconvolution showing the cellular composition with the maximum value for each spot. Each spot represents a spatial location on the tissue, and colors indicate different cell types. **(I)** Spearman correlation between gene expression and microenvironment components at single-cell resolution. The heatmap shows the correlation values, with blue indicating negative correlation and red indicating positive correlation. **(J)** Bar plot illustrating the differential expression of PIGF in malignant regions, mixed malignant regions, and normal regions. The *y*-axis represents the expression level of PIGF, and the *x*-axis categorizes the regions into malignant, mixed, and normal. **(K)** Heatmap of PIGF expression across different tumor stages in OV. The color gradient indicates the expression levels, with red representing higher expression and blue representing lower expression. **(L)** Active landscape of core gene set scores in microzones. The plot shows the activity scores of core gene sets in different microzones, with colors indicating varying levels of gene set activity. **(M)** Bar plot illustrating the differential expression of AUC scores in malignant regions, mixed malignant regions, and normal regions. All these spatial transcriptomics data were sourced from the GSE181300 dataset in GEO (https://www.ncbi.nlm.nih.gov/geo).

Our study provides an in-depth analysis of PIGF gene expression in HNSCC using single-cell sequencing data. Figures 7A–C present UMAP plots displaying major cell lineages (A), single-cell PIGF gene expression (B), and density contour lines (C) in HNSCC, highlighting the distribution and expression patterns of PIGF across different cell populations within the tumor microenvironment. Figures 7D–F show UMAP plots depicting differential expression of core genes across various cell types in HNSCC, illustrating the variability in gene expression among different cell populations. Figure 7G represents pathway differences between core gene-positive and core gene-negative groups across various cell types, with a dot plot displaying enriched pathways in each cell type, indicating functional pathways associated with PIGF expression. Figure 7H utilizes spatial transcriptomic deconvolution to show cellular composition with the maximum value for each spot, providing a spatial map of cell distribution within the tumor. Figure 7I illustrates the Spearman correlation between gene expression and microenvironment components at single-cell resolution, revealing interactions between gene expression and the tumor microenvironment. Figure 7J demonstrates the differential expression of PIGF in malignant, mixed malignant, and normal regions, suggesting its potential role in tumor progression. Figure 7K shows PIGF expression across different tumor stages allowing comparison across various stages of tumor development. Figure 7L presents the active landscape of core gene set scores in microzones, identifying regions within the tumor with high or low activity of specific gene sets. Figure 7M shows differences in AUC scores of gene sets between malignant, mixed malignant, and normal microzones, highlighting significant differences in gene set activity among the different regions. This detailed single-cell sequencing analysis reveals the complex role of PIGF in the tumor microenvironment of HNSCC. Additionally, *in vitro* cell experiments demonstrates that silencing PIGF affects cell proliferation, apoptosis, and the expression of related factors, suggesting potential molecular targets for the HNSCC treatment.

### 3.8 The impact of PIGF on HNSCC cell proliferation, colony formation, and immunotherapy drug sensitivity

**FIGURE 8:**
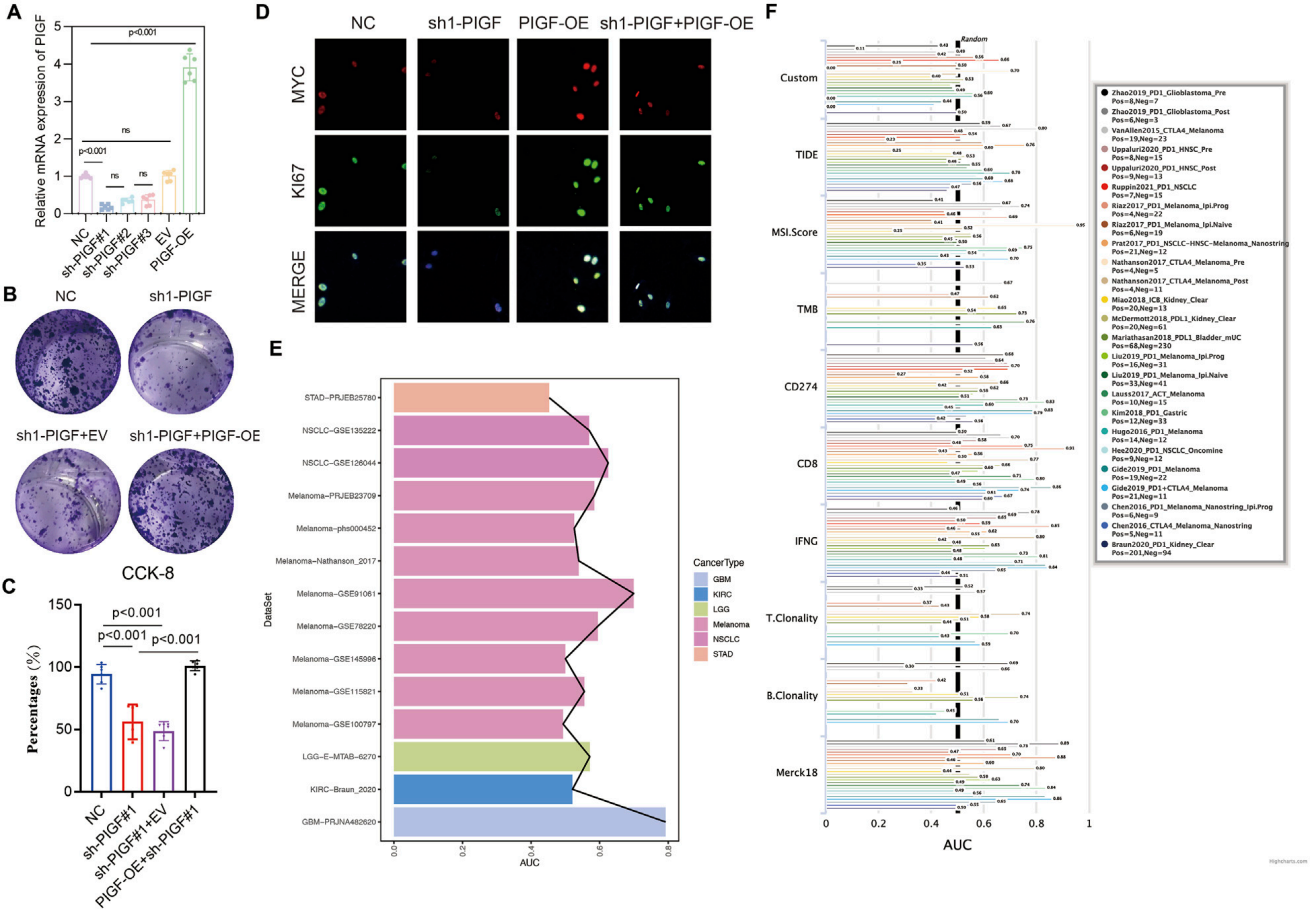
Impact of PIGF Expression on Cell Proliferation and Colony Formation in HNSCC Cell Lines. **(A)** The relative mRNA expression of Placenta Growth Factor (PIGF) in different experimental groups: Negative Control (NC), sh1-PIGF (PIGF knockdown), sh1-PIGF+EV (empty vector), and PIGF-OE (PIGF overexpression). The results indicate a significant decrease in PIGF expression in the sh1-PIGF group compared to the NC and other groups, confirming the efficiency of PIGF knockdown and overexpression constructs. Data are presented as mean ± standard deviation (SD) with statistical significance denoted by ns (non-significant) or specific p-values (p < 0.001). **(B)** Representative images showing the colony-forming ability of colorectal cancer cells under different treatments: NC, sh1-PIGF, sh1-PIGF+EV, and sh1-PIGF+PIGF-OE. Cells are stained with crystal violet, and colonies are visually assessed. Knockdown of PIGF (sh1-PIGF) results in a marked reduction in colony numbers compared to the NC, while overexpression of PIGF (PIGF-OE) reverses this effect, demonstrating the role of PIGF in promoting cell proliferation. **(C)** NC, sh1-PIGF, sh1-PIGF+EV, and sh1-PIGF+PIGF-OE. The results, expressed as a percentage of cell viability, show that PIGF knockdown significantly reduces cell proliferation compared to the control and overexpression groups. Statistical analysis indicates highly significant differences between the groups (p < 0.001). **(D)** Visualization of MYC and Ki67 expression as markers of cell proliferation in colorectal cancer cells. MYC (red) and Ki67 (green) are shown in the NC, sh1-PIGF, PIGF-OE, and sh1-PIGF+PIGF-OE groups. The merged images depict the overall expression pattern, highlighting reduced MYC and Ki67 expression in the sh1-PIGF group compared to others, consistent with decreased cell proliferation and tumor growth potential upon PIGF silencing. **(E)** Predictive performance of PIGF across multiple datasets, with notable variation in its predictive efficacy. PIGF shows strong predictive performance in the PIGF_GBM-PRJNA482620 dataset. **(F)** A forest plot depicting the performance of PIGF-associated models across various cancer types and immunotherapy-related metrics, such as TIDE, MSI score, TMB, CD274 expression, and CD8 infiltration. Variability in performance is noted, with certain datasets showing enhanced predictive accuracy for PIGF’s role in tumor immunology.

This study evaluated the role of PIGF in the proliferation and colony formation of HNSCC cells, as well as its potential as a drug target. By knocking down (sh1-PIGF) or overexpressing (PIGF-OE) PIGF in different experimental groups, real-time quantitative PCR data indicated a significant reduction in PIGF mRNA expression in the sh1-PIGF group, while the PIGF-OE group showed a notable increase, confirming the efficiency of both knockdown and overexpression constructs (Figure 8A). Functional analysis revealed that colony formation in the sh1-PIGF group was significantly reduced, suggesting that PIGF knockdown inhibited cell proliferation. Conversely, PIGF-OE reversed this effect, further demonstrating the critical role of PIGF in promoting HNSCC cell proliferation (Figure 8B). Similarly, the CCK-8 assay showed that PIGF knockdown significantly reduced cell viability, while PIGF overexpression restored the proliferative ability of the sh1-PIGF group (Figure 8C). Immunofluorescence staining demonstrated that the expression of proliferation markers MYC and Ki67 was markedly reduced in the sh1-PIGF group, whereas both markers were upregulated in the PIGF-OE group, supporting the hypothesis that PIGF promotes tumor cell growth by regulating proliferation-related pathways (Figure 8D). To further explore the potential of PIGF as a drug target, we analyzed its predictive performance across multiple cancer datasets (Figure 8E). In the GBM dataset, PIGF exhibited strong predictive power, particularly in the PIGF_GBM-PRJNA482620 dataset. Moreover, analysis of several cancer immunotherapy-related metrics (e.g., TIDE, MSI score, TMB, CD274 expression, CD8 infiltration, IFNγ expression) revealed significant variability in PIGF’s predictive performance across different datasets, with high predictive accuracy observed in glioblastoma and certain melanoma datasets (Figure 8F). These findings suggest that PIGF may play a role in modulating the immune microenvironment, making it a potential target for cancer immunotherapy.

## 4 Discussion

By considering various bioinformatics analysis and cellular experiments, this research revealed a cluster of genes related to exercise for HNSCC prognosis. Specifically, we focus on the PIGF gene, which exhibits significant differential expression in SCC4 cells of HNSCC and shows a close association with tumor diagnostic efficacy, immune therapy response, and immune cell infiltration levels. These findings provide new perspectives on the molecular mechanisms underlying HNSCC and could inform the development of novel therapeutic strategies. The relationship between PIGF gene expression and the tumor immune microenvironment is a significant discovery of this study. We observe that PIGF expression levels are significantly associated with the infiltration degree of various immune cell subsets. Single-cell sequencing analysis further elucidates the complex role of the PIGF gene in the tumor microenvironment, revealing its multifaceted involvement in HNSCC progression and highlighting its potential as a therapeutic target.

The study underscores the importance of understanding gene expression and regulatory mechanisms in diseases contexts, providing an essential basis for understanding the occurrence and development of diseases (Mei et al., 2024). As the critical ontogeny of protein-protein interaction networks and their alterations involved in biological systems have been extensively investigated, they play a crucial role in cell signaling as well as function regulation (Wu et al., 2024; Wu et al., 2023). Meanwhile, discovering gene expression along with its regulatory schemes has brought new insights into deciphering disease complexity of onset or progression where much attention deserves to be paid (Chen N. et al., 2024; Ren et al., 2023). Single-cell RNA sequencing has illuminated cellular response mechanisms in diverse environments (Liu Q. et al., 2023). These studies not only elucidate disease mechanisms but also supply a strong theoretical framework and experimental evidence for subsequent therapies (Gao et al., 2024). Adopting the multi-omics approaches, such as chemical proteomics, deep learning and bioinformatics technologies combined with multiple methodologies could achieve a better understanding of disease mechanism and more accurate development of therapeutic strategies (Liu W. et al., 2024). The long-term analysis of gene expression and regulatory mechanisms in diseases is an indispensable prerequisite for understanding the pathogenesis and progress of a disease (Wong et al., 2023; Monsour and Borlongan, 2023). Through the analysis of pharmacological action, multi-omics data is integrated with advanced bioinformatics techniques to uncover potential therapeutic drug effect and further elucidate the mechanisms of action for drugs; this lays a stable theoretical foundation in clinical application and research. Polymorphism and disease association have been the mainstay of in-depth study for role determination, gene expression as well regulatory mechanisms which are very crucial info with respect to pathologic genome action on about onset and progression of a pathological state (Liu L. et al., 2024). As long as these strategies are fully invested, they will not only improve the scientific depth and breadth of research but also lay a solid foundation for follow-up clinical applications (Zhang J-F. et al., 2023). Validation of effects *in vivo* through animal models and by observation of histopathological changes using histology and immunohistochemical analysis provide important support for further research (Xing et al., 2024). Animal models are essential for studying drug efficacy, and validated animal models can simulate the pathophysiological processes of diseases (Qin et al., 2023). The continuous study of gene expression and regulatory mechanisms in diseases provides new insights into disease progression and therapeutic response by analyzing the immune microenvironment and tumor heterogeneity (Liu J. et al., 2024).

Among these PTMs, the upregulation of placental growth factor (PIGF) has been paid particular attention for its function associated with immune microenvironment in HNSCC (Albonici et al., 2019; Zhang and Han, 2020). PLGF is a member of the vascular endothelial growth factor (VEGF) family and plays roles in angiogenesis, immune cell recruitment, modulation of inflammatory responses as well as tumor immune evasion. Recent bioinformatics analyses highlight PIGF as a potential driver, and biomarker of response in HNSCC spreading its role from tumorigenesis to treatment responsivity. PIGF is a member of the platelet-derived growth factor family encoded by exons 1 and/or 2, which have been described to be modulated production thanks to alternative splicing forms (Vuorela et al., 1997). PIGF is a ligand that can regulate angiogenesis, cell proliferation and migration or invasion in various physiological and pathological settings (De, 2012; Yoo et al., 2015). PIGF plays an important role in the development of a variety of cancers, including oncology studies (Zou et al., 2022). PIGF stimulates tumor cell proliferation, migration and invasion through the binding of its receptor PDGFR-α which leads to downstream signaling pathways activation and also promotes the formation vascular system that will supply nutrients and oxygen needed by tumor (Wang et al., 2023). Another study suggested that PIGF was related to the ability of a tumor cell to evade immune responses and resistance against therapeutic interventions. The expression level of PIGF in HNSCC is closely associated with the aggressiveness, lymph node metastasis and prognosis of these tumors. However, the specific mechanisms of PIGF in HNSCC are not yet fully understood and require further research.

We observed varying correlations between PIGF mRNA and other mRNAs, suggesting that PIGF may have multifaceted roles within the tumor microenvironment of HNSCC (Shiah et al., 2021; Bebek et al., 2011). The presence of both positive and negative correlations indicates that PIGF may function synergistically with or in opposition to other genes, reflecting its involvement in different signaling pathways (Carmeliet et al., 2001; Gerritsen et al., 2003). For example, its interaction with immune-related genes could either promote or suppress immune responses, depending on the cellular context. The negative correlation with CD274 (PD-L1), a key immune checkpoint molecule involved in cancer immune evasion, suggests that higher PIGF expression may be associated with lower PD-L1 levels in HNSCC, potentially reducing immune suppression (Mann et al., 2023; Boschert et al., 2020). This finding aligns with previous studies, which have shown that PIGF expression can be inversely related to immune checkpoint proteins, likely due to its role in modulating the immune microenvironment. Notably, miRNAs positively correlated with PIGF, such as hsa-let-7b-5p and hsa-miR-29c-3p, are known for their roles in regulating tumor growth and immune responses (Salehi et al., 2018; Salehi et al., 2020). This indicates broader regulatory mechanisms influencing cell proliferation, migration, and immune cell infiltration. The observed negative correlations with miRNAs such as hsa-miR-30c-5p and hsa-miR-29b-3p suggest that these miRNAs may have tumor-suppressive functions, and their reduced expression, in combination with elevated PIGF levels, could promote tumor progression by enhancing angiogenesis and reducing immune cell infiltration (Kontomanolis et al., 2019; Orso et al., 2020). This underscores the potential of PIGF and its related miRNAs as therapeutic targets to inhibit tumor growth and modulate the immune landscape in HNSCC. These findings suggest that PIGF plays a dual role in HNSCC by influencing both the angiogenic process and immune response. Its complex interactions with various mRNAs and miRNAs highlight its potential as a therapeutic target. Future research should focus on elucidating the exact mechanisms by which PIGF modulates the tumor microenvironment, particularly in the context of its interactions with miRNAs and immune checkpoint pathways.

The open access and interdisciplinary application of the aforementioned studies further promote the development of precision medicine, emphasizing the importance of comprehensive data analyses and multidimensional assessment in modern medicine (Oinaka et al., 2024). Our cellular experiments confirm that silencing PIGF significantly inhibited HNSCC cells viability and invasive ability. MTT assays, colony formation assays, Transwell assays, and scratch assays collectively demonstrate the critical role of PIGF in HNSCC cell growth and metastasis. In Figure 2H, we observed a significant upregulation of PIGF in stage III and IV head and HNSCC, which was linearly correlated with disease severity. However, the expression level of PIGF in stage II was unexpectedly lower than that in stage I, despite the expectation that PIGF expression would gradually increase with tumor progression. Several potential explanations may account for this anomaly. First, stage II tumors may be influenced by unique microenvironmental factors, such as hypoxia, nutrient availability, or fibroblast activity levels, which differ from those in stage I or more advanced tumors. These factors could render the tumor microenvironment less conducive to PIGF expression. Additionally, variations in inflammation and immune responses between stage II and other stages may also contribute to differences in PIGF expression. Second, the sample size effect may play a role, as stage II tumors might have a smaller or more heterogeneous sample population, which could impact the observed expression pattern. Biological variability between tumors at the same stage may also result in differing PIGF expression levels, with stage II tumors potentially comprising subpopulations characterized by lower inherent PIGF expression. Finally, stage II tumors may involve distinct molecular pathways or regulatory mechanisms that influence PIGF expression differently compared to early or late-stage tumors. These genetic or epigenetic alterations might uniquely affect PIGF regulation in stage II, further contributing to the observed expression pattern. Future studies should focus on examining the tumor microenvironment, immune responses, and molecular pathways unique to stage II HNSCC, as well as exploring potential genetic and epigenetic differences that may regulate PIGF expression. Additionally, expanding the sample size and conducting deeper molecular characterization may help elucidate the biological factors contributing to these observed discrepancies.

In recent years, exercise, as a healthy lifestyle, has been shown to be closely related to the prognosis of various cancers (Idorn and Thor Straten, 2017). Exercise can improve the overall health of patients, enhance immune function, and reduce chronic inflammation, potentially lowering cancer risk and improving cancer patient prognosis (Thomas et al., 2021; Hojman, 2017). Moderate exercise has been shown to benefit HNSCC patients by improving quality of life, alleviating treatment-related side effects, and enhancing survival rates (Avancini et al., 2023). However, the mechanisms by which exercise influences the occurrence and development of HNSCC and whether exercise-related genes exist remain unclear. Investigating the relationship between exercise and HNSCC prognosis, as well as the role of exercise-related genes in HNSCC, is crucial for developing new preventive and therapeutic strategies (Sun et al., 2020; Jin and Yang, 2019). Our study successfully identifies exercise-survival prognosis-related genes in HNSCC and conducts multi-gene analysis. This analysis not only validates the prognostic value of individual genes but also demonstrates the potential of these genes as a collective in predicting HNSCC prognosis. Overall, the results of this study provide new molecular targets and insights for personalized treatment and prognosis evaluation in HNSCC. In this work, we conducted a comprehensive multi-omics investigation, integrating large-scale genomic, transcriptomic, and proteomic datasets to gain a deeper understanding of the molecular mechanisms underlying the biological processes of interest. Rigorous bioinformatics analyses were performed to ensure the robustness and reliability of our findings. Furthermore, we performed integration of the multi-omics datasets using advanced computational approaches, such as correlation analysis and network-based integration, to gain a comprehensive and coherent understanding of the underlying biological mechanisms. These studies not only deepen our understanding of disease mechanisms but also provide a solid theoretical foundation and experimental evidence for future therapeutic strategies (Zeng et al., 2024). By integrating multiple research methods and technologies, including small molecule compound screening, multi-omics analysis, deep learning, and bioinformatics technologies, scientists are continuously exploring and developing new therapeutic strategies, offering new possibilities for precision medicine and personalized treatment (Yin et al., 2024; Li T. et al., 2024).

## 5 Conclusion

In this study, we identified and analyzed exercise-related genes with prognostic significance in HNSCC through comprehensive bioinformatics and cellular experiments. Among these, the PIGF gene demonstrated notable differential expression in HNSCC tissues, and its association with immune cell infiltration, tumor diagnostic efficacy, and therapeutic response positions it as a key biomarker. Furthermore, PIGF’s role in modulating PTMs such as phosphorylation and ubiquitination underscores its potential in regulating cancer progression and immune evasion. These findings provide new insights into the molecular mechanisms underlying HNSCC and suggest that targeting PTMs in PIGF could open novel therapeutic avenues. Future research should further explore the mechanistic roles of PTMs in PIGF regulation, advancing personalized treatment strategies for HNSCC patients.

## Data Availability

The original contributions presented in the study are included in the article/Supplementary Material, further inquiries can be directed to the corresponding author.

## Supplementary material

SUPPLEMENTARY FIGURE 1 PIGF Gene Survival Prognosis Analysis Supplementary. **(A–D)** Independent prognostic analysis to observe whether the association between the PIGF gene and tumor survival is independent of traditional clinical variables, such as age, gender, grade, stage, tumor purity, lymphocyte infiltration, and other factors were considered. Statistical methods used include Univariate Cox (UniCox) and Multivariate Cox (MultiCox) regression analyses. Hazard ratios (HR) with 95% confidence intervals (CI) and p-values are reported for each variable. **(E)** PIGF expression versus survival status plot. The top plot displays PIGF expression levels in high and low-expression groups, while the bottom plot correlates these groups with overall survival status (alive or dead) **(F)** Chi-square test to examine the distribution of survival status (alive vs. dead) across different expression quartiles (Q1-Q4) of PIGF. The p-value indicates the significance of the observed distribution differences. **(G–J)** Restricted cubic spline models exploring the non-linear risk association of the PIGF gene with four survival outcomes: overall survival (OS), disease-specific survival (DSS), progression-free interval (PFI), and disease-free interval (DFI). The splines are adjusted for relevant covariates, and the plots show the estimated log hazard ratios with 95% confidence bands across the range of PIGF expression. **(K–N)** Kaplan-Meier survival analysis for the four survival outcomes: OS, DSS, PFI, and DFI, stratified by high and low PIGF expression groups. Survival curves are presented with log-rank test p-values to assess the statistical significance of the differences between groups. Each plot shows the number of patients at risk over time and the median survival times.
