# Supplementary figures and images for: Targeting exercise-related genes and placental growth factor for therapeutic development in head and neck squamous cell carcinoma
Source: Front Pharmacol. 2024 Oct 4;15:1476076. doi: 10.3389/fphar.2024.1476076 (PMC11486741; doi:10.3389/fphar.2024.1476076)

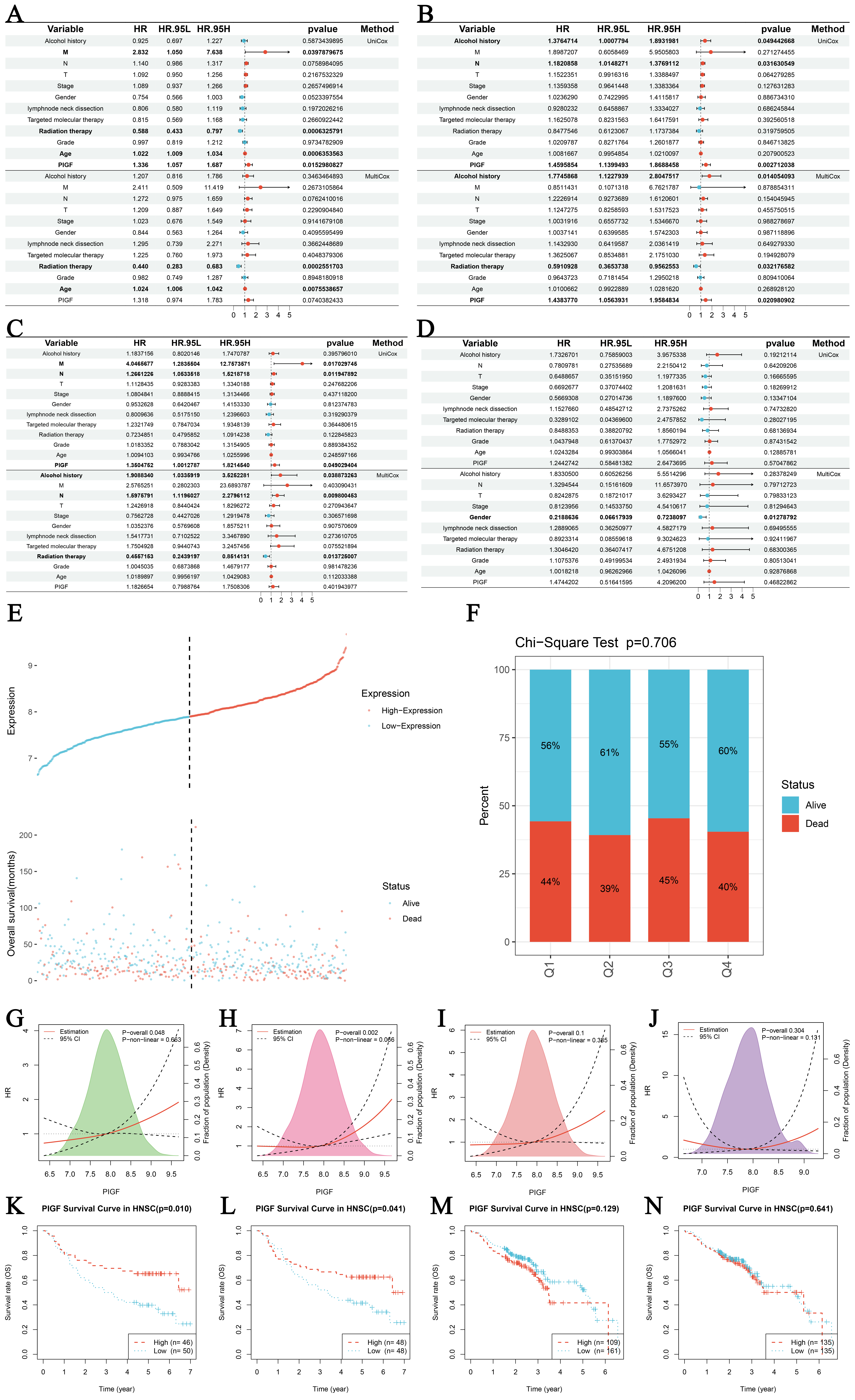

Supplement: Supplementary file 1 [file Image1.PNG]
