# Supplementary material for: Targeting exercise-related genes and placental growth factor for therapeutic development in head and neck squamous cell carcinoma
Source: Front Pharmacol. 2024 Oct 4;15:1476076. doi: 10.3389/fphar.2024.1476076 (PMC11486741; doi:10.3389/fphar.2024.1476076)
Supplement: Supplementary file 2 [file DataSheet1.docx]

**Supplementary Methods**

**Spatial Transcriptomics Analysis**

All spatial transcriptomics data were obtained from the GSE181300 dataset available on GEO (https://www.ncbi.nlm.nih.gov/geo). To evaluate the cellular composition at each spot on the 10x Visium slides, we employed deconvolution analysis, integrating spatial transcriptomics with single-cell transcriptomics data, particularly considering the corresponding cancer types. We collected scRNA-seq data from various instances of the same cancer type to construct a single-cell RNA reference library. To ensure the reliability of the results, we implemented stringent quality control measures based on gene count, UMI counts, and the percentage of mitochondrial RNA, with screening parameters determined in accordance with related studies. A signature score matrix was constructed by calculating the average expression levels of the top 25 specifically expressed genes at each spot. The enrichment score matrix was generated using the Cottrazm package, and the enrichment scores for each cell type were visualized using the SpatialFeaturePlot function from the Seurat package. Higher scores are represented by deeper colors, indicating a greater abundance of that cell type. A score of 1 was defined as the Malignant group, 0 as the Normal group, and all other scores as the Mixed group. The statistical significance of specific gene expression differences between the three groups was assessed using the Wilcoxon rank-sum test.
